# Feeding challenges faced by Brazilian children and adolescents with infantile neuroaxonal dystrophy (INAD) adversely impact nutrient intake and nutritional status

**DOI:** 10.31744/einstein_journal/2026AO1914

**Published:** 2026-09-09

**Authors:** Laura Fantazzini Grandisoli, Danilo de Assis Pereira, Natália Cristina Marques Spuzzillo, Verônica Leite Sakalauskas, Luis Armando Souza Vieira, Maria Eduarda Munhoz Simeão Baganha, Mariana Battaglin Villas Boas Álvaro, Reinaldo José Gianini

**Affiliations:** 1 Universidade Paulista Sorocaba SP Brazil Universidade Paulista, Sorocaba, SP, Brazil.; 2 Pontifícia Universidade Católica de São Paulo Department of Human Reproduction and Childhood Sorocaba SP Brazil Department of Human Reproduction and Childhood, Pontifícia Universidade Católica de São Paulo, Sorocaba, SP, Brazil.; 3 Universidade Estadual de Campinas Postgraduate Program Campinas SP Brazil Postgraduate Program, Universidade Estadual de Campinas, Campinas, SP, Brazil.

**Keywords:** Neuroaxonal dystrophies, Infant, Neurodegenerative diseases, Deglutition disorders, Nutritional status, Enteral nutrition

## Abstract

**Objective:**

To evaluate the nutritional profile, dietary intake, and feeding characteristics of Brazilian children and adolescents with infantile neuroaxonal dystrophy, comparing exclusively orally fed and gastrostomy-fed participants.

**Methods:**

This retrospective cross-sectional study included 21 genetically confirmed patients with infantile neuroaxonal dystrophy between July 2023 and March 2024. Data collection involved 24-hour dietary recalls, anthropometric measurements, and assessment of dysphagia using the Functional Oral Intake Scale. Nutritional status and intake were compared using the Mann-Whitney U test (p<0.05).

**Results:**

Twelve participants (57%) received gastrostomy feeding. Among orally fed participants (n=9), 66.7% required food texture modifications and 88.9% exhibited symptoms suggestive of dysphagia. Weight-for-age Z-scores tended to be lower in the exclusively orally fed group than in the gastrostomy-fed group (−2.10 *versus* −0.34, p=0.072). Micronutrient intake, including vitamins A, D, E, folic acid, and B12, was lower in the exclusively orally fed group than in the gastrostomy-fed group. Ultra-processed foods were frequently consumed, leading to a monotonous diet.

**Conclusion:**

Feeding difficulties are common among patients with infantile neuroaxonal dystrophy and are associated with impaired nutritional intake and suboptimal growth, particularly in exclusively orally fed individuals. These findings underscore the importance of individualized nutritional strategies and multidisciplinary care to address dysphagia and optimize nutritional outcomes.

## In Brief

Grandisoli et al. evaluated 21 Brazilian patients with infantile neuroaxonal dystrophy and identified a high prevalence of dysphagia and feeding difficulties. The exclusively orally fed group exhibited significantly lower intake of vitamins A, D, E, B9, and B12, alongside lower weight-for-age Z-scores than that in the gastrostomy-fed group. The study underscores the need for multidisciplinary feeding and nutritional management in patients with infantile neuroaxonal dystrophy.

## Highlights

Frequent dysphagia in infantile neuroaxonal dystrophy may result in poor nutritional outcomes.

Most children required texture modification or enteral feeding.

Orally fed participants consumed monotonous diets high in ultra-processed foods.

Orally fed patients exhibit an increased risk of micronutrient deficiencies.

## Graphical abstract

**Figure:**
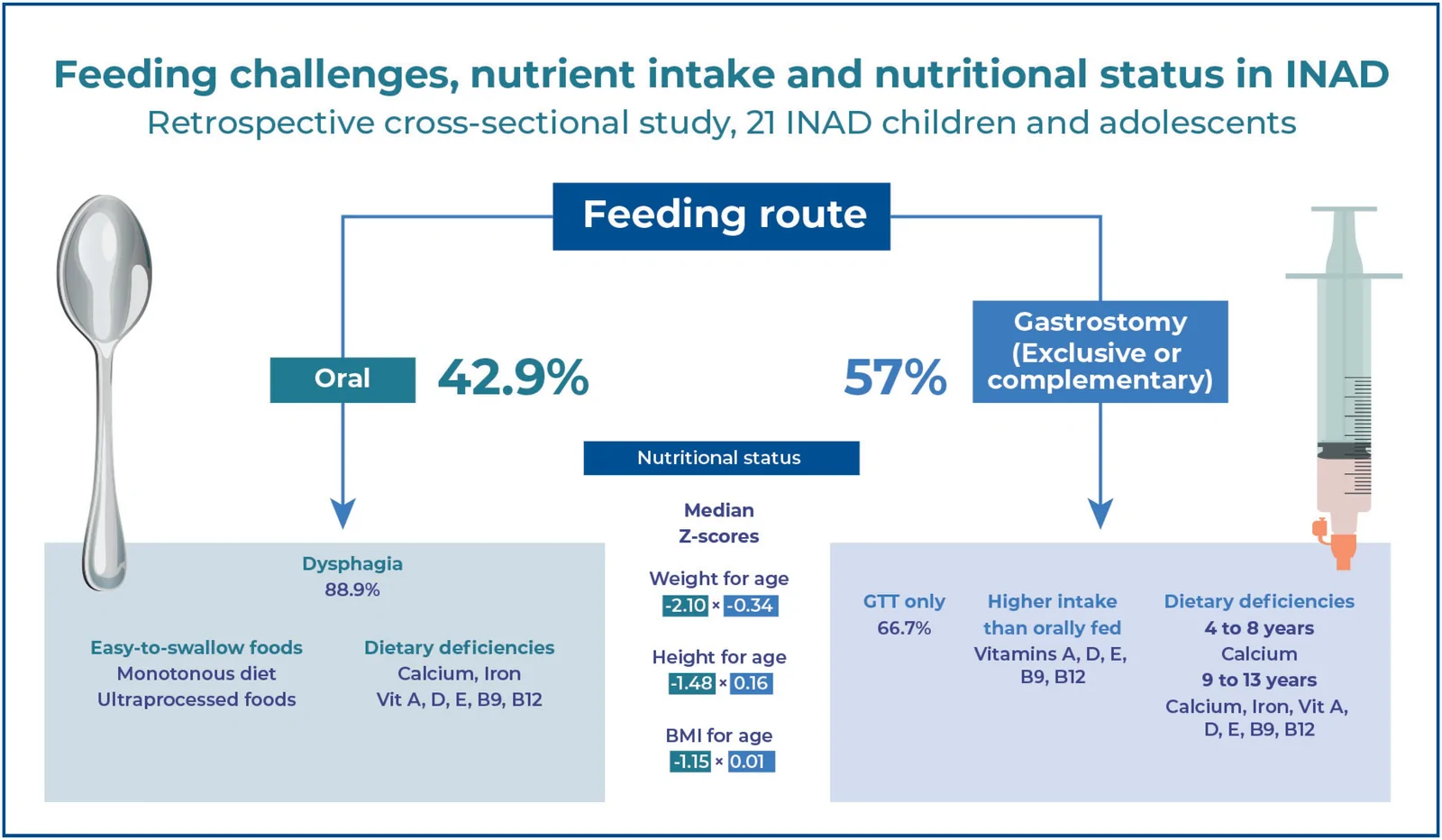

## INTRODUCTION

Infantile neuroaxonal dystrophy (INAD) is an ultra-rare, severe neurodegenerative disorder classified as a subtype of PLA2G6-associated neurodegeneration (PLAN).^(1-4)^ PLA2G6-associated neurodegeneration comprises several clinical subtypes, including INAD and atypical neuroaxonal dystrophy, and PLA2G6-related dystonia-parkinsonism, each characterized by distinct phenotypic features.^(3,5,6)^ The *PLA2G6* gene, located on chromosome 22q13.1, encodes a crucial enzyme essential for phospholipid metabolism. Dysfunction of this enzyme impairs membrane repair mechanisms and contributes to progressive neuronal damage.^(1-4)^

Infantile neuroaxonal dystrophy typically manifests between 6 months and 3 years of age. Early clinical features include psychomotor regression, gait disturbances, and progressive spastic tetraparesis.^(1,6)^ The disease progresses rapidly and is commonly associated with severe cognitive decline, optic atrophy, and bulbar dysfunction, leading to early mortality, often before the second decade of life.^(1,6-8)^

As the disease progresses, swallowing function becomes increasingly impaired because of neurological deterioration, neuromuscular junction dysfunction, and muscle atrophy, resulting in dysphagia.^(1,6-8)^ Dysphagia may subsequently contribute to pulmonary complications and nutritional deficiencies. Impaired swallowing efficiency compromises the transfer of food from the oral cavity to the esophagus, thereby increasing the risk of malnutrition and dehydration. In addition, swallowing dysfunction may lead to aspiration, where saliva, liquids, or food enter the airways, posing additional health risks.^(9,10)^

Given the complexity of INAD, maintaining adequate nutritional intake is essential. Management strategies commonly include food texture modification and nutritional supplementation to address the specific needs of affected children. As feeding difficulties and nutritional issues worsen with disease progression, gastrostomy tube feeding is often recommended to ensure adequate nutritional support.^(11,12)^

Despite the critical importance of nutritional care in INAD, studies comprehensively evaluating dietary adequacy and nutritional status in children and adolescents with this condition remain lacking. A better understanding of the nutritional and feeding challenges experienced by patients with INAD is crucial for developing effective nutritional management strategies aimed at improving quality of life.

## OBJECTIVE

We aimed to characterize the nutritional profile, dietary intake, and associated conditions, such as dysphagia, among orally and tube-fed Brazilian patients with infantile neuroaxonal dystrophy.

## METHODS

### Study Design and Participants

This retrospective cross-sectional study was conducted between July 2023 and March 2024. Owing to the rare nature of the condition studied, a formal sample size calculation was not performed. Instead, a convenience sampling strategy was adopted, including all eligible individuals identified during the study period. Participants were recruited through a social network support group for families of children and adolescents with INAD. Eligibility criteria included a compatible medical history, molecular confirmation of INAD diagnosis through genetic testing, and provision of written informed consent. Although the study primarily included genetically confirmed cases of INAD, limitations related to the interpretation of variants of uncertain significance and other potential diagnostic confounders are acknowledged.

Individuals with hereditary movement disorders without molecular confirmation of PLA2G6-related INAD, asymptomatic carriers, and those unable to provide consent were excluded. After application of the eligibility criteria, clinical and examination data from 21 patients aged 3-21 years were included in the analysis.

### Data collection

Caregivers completed a semi-structured electronic questionnaire that collected information on sociodemographic data, neuropsychomotor developmental milestones, anthropometric parameters (weight and height), multidisciplinary health support, supplement use, and signs suggestive of dysphagia, including coughing, choking, wet voice, and prolonged feeding times. Moreover, to improve data accuracy and completeness, caregivers also participated in telephone interviews to confirm patient histories. In addition, caregivers submitted serum test results and genetic testing documentation for further analysis.

### Nutritional status and dietary intake assessment

Nutritional status was assessed using reported body weight and height measurements to calculate body mass index (BMI). Weight-for-age Z scores (WAZ), height-for-age Z scores (HAZ), and BMI-for-age Z-scores (BMIAZ) were calculated according to the World Health Organization (WHO) child growth standards.^(13,14)^ Z-scores were generated using WHO Anthro and WHO Anthro Plus software programs.^(15,16)^

Dietary intake was evaluated using three non-consecutive 24-hour dietary recalls administered to caregivers by trained dietitians via telephone or video calls, including one recall collected on a weekend day. The multiple-pass method was used to improve accuracy.^(17,18)^ Caregivers reported all foods and beverages consumed, including homemade preparations, with detailed descriptions of ingredients, cooking methods, food texture, and portion sizes. For patients receiving industrial nutritional supplements or enteral nutrition formulas, information regarding product brand, type, and daily intake volume was collected. To enhance accuracy, a photographic food quantification manual containing standardized household measures, food preparation guidelines, and images of typical food portion sizes was used. All portion sizes were standardized to grams for solid foods and milliliters for liquids.^(19-23)^

Nutritional analysis of foods and beverages was performed using the Diet Box^^®^^ online software, which incorporates data from Brazilian food composition databases.^(24-26)^ Mean energy and nutrient intakes were calculated from the three dietary recalls. Micronutrient analysis focused on calcium, iron, zinc, and vitamins A, D, E, B9, and B12 because deficiencies in these nutrients are highly prevalent in Brazil.^(27)^ Median micronutrient intake values were compared with the age-specific Dietary Reference Intake guidelines from the Institute of Medicine.^(28)^ Given the heterogeneity of the sample, individual reference standards, including the Recommended Dietary Allowance (RDA) or adequate intake (AI), were used instead of population-based reference values, such as the Estimated Average Requirement.

To assess food group consumption frequency, each portion consumed during each recall was categorized into the following groups: ultra-processed foods (e.g., chocolate drinks, sugary drinks, flavored yogurts, cake with toppings, stuffed biscuits, mayonnaise, chocolate); cereals, roots, and tubers; fruits; vegetables; milk and dairy products; meats and eggs; legumes; nuts and seeds; food supplements; and added sugar.^(29)^

Although caregivers were not directly questioned regarding feeding classification, each participant's feeding method was retrospectively categorized using the Functional Oral Intake Scale (FOIS) for children, as follows: Level 1, nothing by mouth; Level 2, tube-dependent with minimal oral intake (minimal attempts or a very small amount of oral intake); Level 3, combined tube and oral feeding with consistent oral intake contributing significantly to caloric intake; Level 4, total oral diet requiring special preparation or compensatory strategies such as food grinding, fluid thickening, or use of nutritional supplements, or limited to exclusive bottle feeding; and Level 5, total oral diet without special preparation or compensations.^(30)^

### Ethical considerations

This study received approval from the Research Ethics Committee of the *Pontifícia Universidade Católica de São Paulo* (CAAE: 65887222.0.0000.5373; # 7.598.226). Written informed consent was obtained from all legal guardians before study inclusion.

### Statistical analysis

Statistical analyses were performed using IBM SPSS Statistics version 20.0.^(31)^ Continuous variables were assessed for normality using the Shapiro-Wilk test and were found to have a non-normal distribution. Therefore, parametric tests such as the Student's t-test or analysis of variance (ANOVA) were considered inappropriate because their underlying assumptions of normality and homogeneity of variance were not met. Accordingly, non-parametric methods were applied. Medians and interquartile ranges (IQR) (25th and 75th percentiles) were reported as measures of central tendency and dispersion, respectively, and group comparisons were performed using the Mann-Whitney U and Kruskal-Wallis tests, as appropriate.

Furthermore, owing to the small overall sample size and even smaller subgroups, regression analyses, either simple or multiple, were not conducted, as they would likely produce imprecise estimates with wide confidence intervals, thereby limiting the interpretability and reliability of the findings.

## RESULTS

### Sociodemographic and Clinical Characteristics of Participants

The study included 21 participants, with a predominance of male individuals. The median age (IQR) was 8.2 years (5.5-10.0). Consanguinity was reported in nearly one-third of the sample. Most mothers had completed higher education, and most families had health insurance coverage (Table 1).

**Table 1:** Sociodemographic and clinical characteristics of participants (n=21)

Variables		Value	p value
Age, median (IQ range), years		7.0 (5.5–10.0)	-
Sex, n (%)			
	Male	14 (66.7)	0.127
	Female	7 (33.3)	
Ethnicity, n (%)			
	White	17 (81.0)	0.005*
	Mixed	4 (19.0)	
Parents consanguinity, n (%)			
	Yes	6 (28.6)	0.050*
	No	15 (71.4)	
Mother's age in labor, median (IQR), years		32 (24–36)	-
Mother's education, n (%)			
	Complete or incomplete primary education	2 (9.5)	0.004*
	Complete or incomplete secondary education	5 (23.8)	
	Undergraduate/graduate/postgraduate	14 (66.7)	
Monthly family income, n (%)			
	<BRL 2.000,00 (≤1.5 minimum wage)	5 (23.8)	0.180
	BRL 2.001,00–5.000,00 (1.5–3.5 minimum wage)	11 (52.4)	
	>BRL 5.000,00 (3.5 minimum wage)	5 (23.8)	
Health insurance, n (%)			
	Yes	18 (85.7)	0.001*
	No	3 (14.3)	
Child education, n (%)			
	Currently attending school	7 (33.3)	0.867
	Previously attended school but no longer does	6 (28.6)	
	Never attended school	8 (38.1)	
Physical disability, n (%)			
	Loss of neck control	16 (76.2)	-
	Loss of trunk control	17 (81.0)	
	Loss of walking ability	20 (95.2)	
	Scoliosis	12 (57.1)	
	Dislocated hip	5 (23.8)	
	Ankle deformity	9 (42.9)	
	Gastrointestinal disorders	13 (61.9)	
Ophthalmological abnormalities		16 (76.2)	
Tracheotomy		3 (14.3)	
Gastrostomy		12 (57.1)	
Feeding route, n (%)			
Exclusive oral feeding		9 (42.9)	0.368
Exclusive enteral feeding		8 (38.1)	
Combined enteral and oral feeding		4 (19.0)	
FOIS classification, n (%)			0.422
	Level 2	8 (38.1)	
	Level 3	4 (19.0)	
	Level 4	6 (28.6)	
	Level 5	3 (14.3)	

Statistical significance was defined as p<0.05.

* Difference is statistically significant.

IQR: interquartile range; BRL: Brazilian Real; FOIS: Functional Oral Intake Scale.

Among the 21 participants, significant physical disabilities were prevalent. Most patients exhibited loss of walking ability, trunk control, and neck control. Gastrointestinal manifestations were also prevalent (61.9%) and included constipation, reflux, and abdominal distention (Table 1).

A large proportion of participants (57.1%, n=12) required an alternative feeding route via gastrostomy, either exclusively or in combination with oral feeding. The classification by the FOIS for children demonstrated that most participants were classified as Level 2, indicating tube dependence with minimal oral intake, followed by Level 4, indicating an exclusive oral route requiring special preparation of food textures or compensatory strategies. Only three patients were classified as FOIS Level 5, corresponding to total oral diet without special preparations or compensatory strategies. However, two of these patients exhibited signs of dysphagia according to caregiver reports (choking and coughing), which may indicate inadequacies between food texture and swallowing capacity (Table 1).

### Feeding characteristics of exclusively orally fed participants

Among the nine participants who were exclusively orally fed, six (66.7%) required some degree of food texture modification. Dysphagia symptoms were prevalent, with 88.9% of patients exhibiting at least one sign of swallowing dysfunction. The most frequently reported symptom was choking during meals, followed by coughing while eating, prolonged meal duration exceeding 30 minutes, and a wet voice during feeding. Despite these challenges, a significant portion of participants lacked specialized professional support. One-third of participants did not receive assistance from a speech-language pathologist, and nearly half had no dietitian assistance (Table 2).

**Table 2:** Feeding characteristics and specialist support of exclusively orally fed participants (n=9)

Variables		Value
Food texture, n (%)		
	Same consistency as family meals	3 (33.3)
	Mashed/shredded texture	3 (33.3)
	Blended texture	3 (33.3)
Signs suggestive of dysphagia, n (%)		
	Choking during meals	7 (77.8)
	Coughing during meals	4 (44.4)
	Meal duration lasts longer than 30 minutes	4 (44.4)
	Wet voice during feeding	1 (11.1)
	Presence of at least one dysphagia-related sign	8 (88.9)
Speech-language pathologist assistance, n (%)		6 (66.7)
Dietitian assistance, n (%)		5 (55.6)

### Anthropometric outcomes

Based on BMI assessments, most participants (75%; n=15) were classified as having normal weight. Among the remaining participants, one was classified as overweight, two as obese (including one patient classified as having severe obesity), and two were underweight (including one patient classified as severely underweight). Participant 17 could not be classified because weight and height data were unavailable. In addition, Participant 12 was 21 years old and was therefore classified according to adult BMI cut-off criteria. Figure 1 illustrates the BMIAZ scores for each participant.

**Figure 1:**
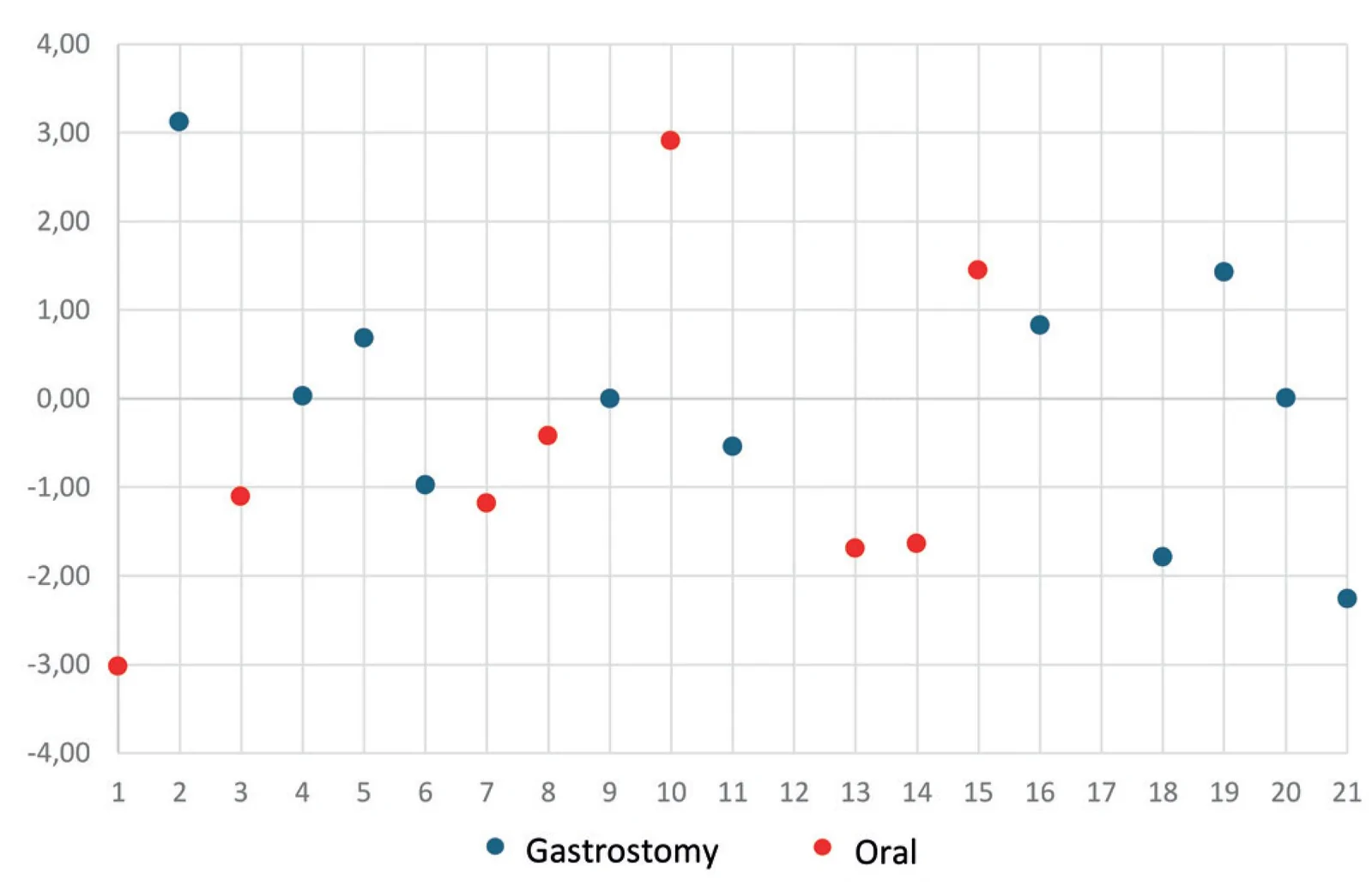
BMI-for-age Z-score participants by feeding route

Table 3 shows anthropometric Z-scores according to feeding route. Participants were categorized into two groups: the exclusively orally fed group (n=9) and the gastrostomy-fed group, either exclusively or in combination with oral feeding (n=12). No significant differences were observed between the groups regarding median age (8 *versus* 6 years, p=0.148), sex distribution (p>0.999), race (p=0.009), or maternal education level (p=0.319) (data not shown).

Median WAZ tended to be lower in the exclusively orally fed group than in the gastrostomy group (−2.10 *versus* −0.34, respectively; p=0.072). Median HAZ and BMIAZ were also lower in the orally fed group than in the gastrostomy group, although these differences did not reach statistical significance (Table 3).

**Table 3:** Z-scores of anthropometric parameters according to the feeding route

Anthropometric parameters	Feeding route		p value
	Exclusive oral feeding (n=9)	Gastrostomy feeding exclusively or complementary (n=10)	
WAZ	−2.10 (−2.83 to −1.42)	−0.34 (−1.46 to 1.63)	0.072
HAZ	−1.48 (−2.41 to −0.03)	0.16 (−1.86 to 1.21)	0.272
BMIAZ	−1.15 (−1.68 to 0.98)	0.01 (−0.97 to 0.83)	0.351

Data presented as median (interquartile range). Mann–Whitney test. Statistical significance was defined as p<0.05.

WAZ: weight-for-age Z-score; HAZ: height-for-age Z-score; BMIAZ: body mass index-for-age Z-score.

### Dietary intake

Analysis of food consumption demonstrated that the exclusively orally fed group had higher intakes of protein and fiber than did the gastrostomy-fed group. However, the intake of lipids and several key vitamins, including vitamins A, D, E, folic acid, and B12, was significantly lower in the exclusively orally fed group than in the gastrostomy-fed group (Table 4).

**Table 4:** Calories and nutrient intake according to the feeding route

Variables	Feeding route		p value
	Exclusive oral feeding (n=9)	Gastrostomy feeding exclusively or complementary (n=12)	
Energy (kcal)	1195.7 (986.2–1590.2)	1125.8 (782.0–1397.9)	0.177
Protein (g)	58.5 (49.8–80.2)	31.2 (24.17–46.9)	0.016*
Protein (g/kg)	3.99 (1.50–4.64)	1.26 (0.63–2.01)	0.023*
Carbohydrates (g)	191.2 (157.0–238.9)	158.9 (104.7–186.1)	0.065
Lipids (g)	25.3 (21.0–42.3)	35.9 (23.0–52.7)	0.032*
Fiber (g)	16.0 (14.5–20.7)	3.9 (0.0–8.8)	0.002*
Calcium (mg)	767.81 (451.80–938.27)	718.5 (503.60–841.80)	0.670
Iron (mg)	8.10 (5.70–9.57)	11.09 (71.16–14.70)	0.070
Zinc (mg)	7.65 (4.50–9.32)	8.90 (5.03–14.95)	0.320
Vitamin A (mcg)	245.53 (186.67–347.33)	437.39 (390.723–692.41)	0.007*
Vitamin D (mcg)	3.5 (0.85–5.67)	10.77 (5.43–15.48)	0.003*
Vitamin E (mg)	2.03 (0.27–4.18)	13.25 (9.38–19.18)	<0.001*
Folic acid (mcg)	0.52 (0.36–0.87)	165.68 (114.37–238.27)	<0.001*
Vitamin B12 (mcg)	0.27 (0.07–0.65)	2.36 (1.30–2.91)	<0.001*

Data presented as median (interquartile range). Mann–Whitney test. Statistical significance was defined as p<0.05.

* Difference is statistically significant.

To assess micronutrient intake adequacy, participants’ dietary intake was compared with the RDA. Results revealed that the gastrostomy-fed group generally had better nutrient adequacy than that in the exclusively orally fed group.

Only zinc intake met recommended levels among participants aged 0 to 8 years in the exclusively orally fed group. For those aged 9 to 13, the intake of all assessed micronutrients was below the RDA (Figure 2).

**Figure 2:**
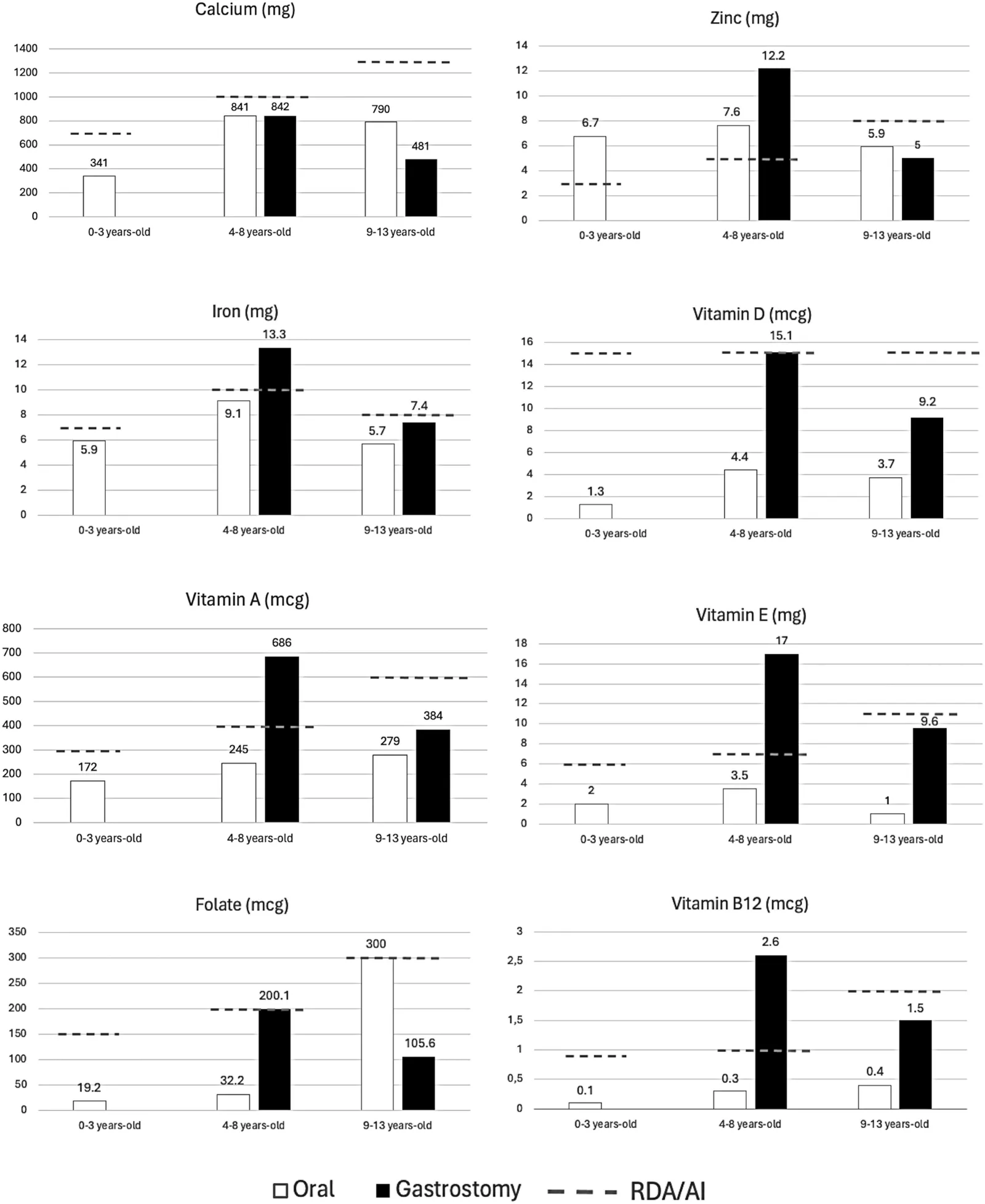
Micronutrient intake according to the route of feeding, against the recommended dietary intake (Recommended Dietary Allowances/Adequate Intake)

Conversely, participants aged 4-8 years in the gastrostomy-fed group met the RDA for all micronutrients except calcium. Zinc intake for this group slightly exceeded the upper limit of 12mg, with a median intake of 12.2mg. However, for participants aged 9-13 years in the gastrostomy-fed group, intake of all micronutrients was below the RDA. No participants aged 0-3 years were recruited in the gastrostomy-fed group (Figure 2).

Analysis of dietary recalls revealed a predominance of ultra-processed food consumption among patients, accounting for 71 reported instances (22.0%), mainly consisting of flavored and sweetened yogurts consumed as snacks, followed closely by cereals, roots, and tubers, with 68 instances (21.1%). Fruits and vegetables were consumed 36 (11.2%) and 33 (10.2%) times, respectively, whereas milk and dairy products accounted for 32 records (9.9%). Meats and legumes were slightly less frequently reported, with 30 (9.3%) and 28 (8.7%) instances, respectively. Other food groups, such as nutritional supplements (12; 3.7%), added sugars (6; 1.9%), eggs (4; 1.2%), and nuts/seeds (2; 0.6%), showed minimal representation. Overall, the pattern reflected a monotonous diet with limited food variety, with frequent repetition of similar foods across meals.

Regarding specific nutrient supplementation, Participants 7, 8, and 15 received omega-3 supplements, whereas only Participant 7 consumed a multivitamin supplement. These supplements were not included in the dietary intake analysis.

## DISCUSSION

Children and adolescents with neuroaxonal dystrophy associated with PLA2G6 gene mutations experience substantial feeding difficulties that may adversely affect nutrient intake and nutritional status. Nearly all participants in the present study exhibited signs suggestive of dysphagia, and most required an alternative feeding route. Among the exclusively orally fed group, signs suggestive of dysphagia were strongly evident, leading to inadequate weight gain and insufficient micronutrient intake, thereby increasing nutritional risk and potentially exacerbating their overall clinical deterioration.

To our knowledge, no previous studies have specifically characterized dysphagia in children with INAD. Based on clinical experience, disease progression may be accompanied by tracheal aspiration, particularly of thin foods and saliva. However, the greatest impact of dysphagia in this population may relate to nutritional compromise, as swallowing dysfunction in INAD is often characterized by marked inefficiency, multiple swallowing attempts to clear pharyngeal residue, and prolonged oral and pharyngeal transit times.

The FOIS classification demonstrated that most participants were categorized as Levels 2 or 4, reflecting varying degrees of dysphagia severity, ranging from mild, requiring only adjustments in food consistencies, to severe impairment requiring exclusive gastrostomy feeding. Additionally, caregivers reported signs suggestive of dysphagia in two of the three participants classified as FOIS Level 5.

Feeding patients with dysphagia safely is complex and requires individualized adjustments in posture, utensils, and food consistencies to facilitate safe swallowing while supporting adequate nutritional and hydration status. Food texture modification is therefore essential to optimize adequate nutrition and reduce aspiration risk. As dysphagia severity progresses, the need for such interventions increases, often leading to the recommendation of alternative feeding routes.

Gastrostomy is generally considered a relatively safe procedure associated with nutritional benefits and reduced risk of pulmonary complications following placement.^(32)^ These clinical benefits may be more pronounced when gastrostomy is indicated before the development of severe respiratory or nutritional impairment, potentially contributing to improved clinical stability, better quality of life, and prolonged maintenance of partial oral feeding. More than half of the children in the study had already undergone gastrostomy for enteral feeding, either exclusively or as a supplement to oral feeding. The exclusively orally fed group demonstrated multiple clinical signs of tracheal aspiration during meals, most commonly choking and coughing. The European Society for Pediatric Gastroenterology, Hepatology, and Nutrition emphasizes the importance of multidisciplinary management for children with neurological impairments, involving occupational therapists, psychologists, speech-language pathologists, dietitians, physicians, and nurses. Such coordinated care may help reduce nutritional deficiencies, improve gastrointestinal outcomes, and reduce aspiration risks.^(12,32)^ Nevertheless, despite these recommendations, one-third of the exclusively orally fed group in this study did not receive speech-language pathology follow-up, and only 55% had access to dietitian support.

Regarding nutritional status, most participants presented with a BMI within the normal range; however, the exclusively orally fed group had lower scores across all anthropometric parameters. Lower body weight was observed among the exclusively orally fed group than in the gastrostomy-fed group (p=0.072), with a median WAZ of −2.1 in the exclusively orally fed group, consistent with moderate underweight status. In contrast, the gastrostomy-fed group presented with a median WAZ of −0.34, corresponding to WA values within the normal range. Although no statistically significant differences were identified for HAZ and BMIAZ, the findings suggest a clinically relevant impact on growth and nutritional status. The small sample size may have reduced statistical power; nevertheless, the observed clinical differences highlight the importance of continuous nutritional monitoring. Research involving patients with cerebral palsy (CP) has demonstrated a strong association between dysphagia, malnutrition, dehydration, and failure to thrive. In addition to dysphagia, these patients frequently exhibit global motor impairments, which further compromise food intake.^(33-35)^ Leonard et al.^(36)^ reported that, among 260 children with CP, 34% were malnourished, and 38% had dysphagia, with dysphagia being associated with more severe nutritional deficits. Similarly, Zhao et al. reported that the likelihood of moderate to severe malnutrition was strongly associated with dysphagia and gross motor impairments in patients with CP.^(35,36)^ A similar pattern may be present in patients with INAD, in whom disease progression is often accompanied by worsening dysphagia over time.

The specific dietary requirements of children and adolescents with INAD remain poorly characterized. Neurological disorders present unique challenges in estimating energy requirements because functional capacity, muscle tone, and physical activity levels change over time.^(11,37)^ Energy requirements may decline as the disease progresses, except in the presence of dystonia, thereby necessitating regular dietary reassessment and adjustment of dietary prescriptions. In contrast, there is not enough evidence suggesting that protein and micronutrient needs are reduced in children with neurological impairments, making it difficult to provide adequate nutrients within a low-calorie diet.^(37)^

Analysis of dietary intake revealed significant differences in macronutrient and micronutrient consumption between the exclusively orally fed and gastrostomy-fed groups. Protein intake should be sufficient to support a child's growth, development, and maintenance of muscle mass. Accordingly, protein requirements vary across age groups, decreasing from approximately 1.2g/kg in infants aged 7-12 months to 0.85g/kg in adolescents aged 14-18 years.^(38)^ Protein intake in the exclusively orally fed group was substantially higher than recommendations, with a median intake of 3.9g/kg, whereas the gastrostomy-fed group exhibited a median intake of 1.2g/kg, close to recommended levels. This disparity indicates an imbalance that necessitates careful monitoring.

Low fiber intake was common in the gastrostomy-fed group, with some children receiving no fiber because of reliance on commercial formulas lacking fiber content. Inadequate fiber intake can exacerbate gastrointestinal issues, particularly constipation, and may negatively affect gut microbiome diversity.^(37,38)^ Potential interventions include the use of fiber-enriched enteral formulas, blenderized feeds, soluble fiber supplementation, and stool softeners.

Micronutrient intake also differed between feeding groups, with the gastrostomy-fed group receiving higher levels of vitamins A, D, E, folic acid, and B12 than those in the exclusively orally fed group. Comparisons between dietary intake and recommended values suggested that the exclusively orally fed group is at a higher risk of micronutrient deficiencies across all age groups. A study by Gomes et al. demonstrated an association between motor function, an individual's feeding competencies, and micronutrient intake.^(39)^ Furthermore, dysphagia-related symptoms during feeding, including choking, coughing, and prolonged mealtimes, have been associated with micronutrient deficiencies.^(39-41)^

Conversely, patients aged 4-8 in the gastrostomy-fed group met daily requirements for all evaluated micronutrients, except calcium. This finding is expected because enteral feeding formulas are generally designed to meet daily micronutrient requirements. However, among patients aged 9-13, intake of all assessed micronutrients remained below recommended levels. Even when complete enteral nutrition formulas are used, micronutrient deficiencies may arise if there is an imbalance between the formula volume required to meet energy requirements and the intake needed to fulfill micronutrient targets, as previously mentioned. In such situations, formula volume and micronutrient supplementation must be adjusted to individual needs.

In addition to motor impairments, limited dietary variety among the exclusively orally fed group also influenced micronutrient intake. The repetitive nature of the diet, characterized by frequent consumption of the same foods, likely reduced dietary variety and micronutrient adequacy. A high intake of ultra-processed foods, particularly flavored yogurts, was observed and accounted for 22% of total reported food intake. Although these foods may be easier to consume, they are generally characterized by high sugar content and low micronutrient density, which may contribute to poor dietary quality. For children with dysphagia, the need for texture-modified diets may further restrict dietary diversity, because preparing foods with safe swallowing consistency often requires considerable effort. Consequently, caregivers may preferentially rely on familiar and easily prepared foods, further limiting dietary variety. Because micronutrient composition varies substantially across food sources, particularly vegetables and fruits, dietary diversification is essential to ensure adequate micronutrient intake. Micronutrient deficiencies may have important consequences for growth, neurodevelopment, immune function, and overall health. Vitamin D plays a central role in calcium homeostasis and bone growth by promoting chondrocyte maturation and endochondral ossification while also modulating immune responses. Zinc is a cofactor for numerous enzymes involved in cell growth and differentiation and contributes to immune, neurological, and reproductive functions, as well as wound healing and taste response. It also has antioxidant and anti-inflammatory properties. Iron deficiency, including iron deficiency anemia, represents one of the most common nutritional issues in children and adolescents worldwide, leading to fatigue, weakness, and impaired physical activity due to reduced levels of hemoglobin, myoglobin, and cytochromes. Iron is also critical for cognition and neurodevelopment.^(42,43)^ Vitamin E functions as a potent antioxidant agent, preventing lipid peroxidation in cell membranes and supporting immune function. Its deficiency, common in Brazil, can result in hemolysis and progressive peripheral neuropathy.^(44,45)^ Vitamin B12 and folic acid deficiencies are primary causes of megaloblastic anemia, a type of macrocytic anemia occurring because of inefficient red blood cell production and hemolysis. Vitamin B12 deficiency can also lead to neuropsychiatric symptoms, such as spinal cord degeneration, memory loss, delirium, depression, and hallucinations.^(46,47)^

Oral feeding remains the preferred approach for children with neurological impairments whenever it can be performed safely and efficiently. Feeding should be safe, stress-free, and provide optimal energy and nutrients within a reasonable feeding duration. In this context, our findings highlight the need for a thorough, individualized, and multidisciplinary evaluation of patients with INAD to ensure growth, identify potential nutrient deficiencies, and support early interventions that enhance health and well-being.

This study has some limitations. First, reported weight and height may influence the accuracy of nutritional status assessments. However, data collection through an online form enabled us to reach a broader patient base across Brazil's vast territory. Second, self-reported dietary intake tools may introduce bias, although 24-hour recalls are generally more accurate than food frequency questionnaires in calculating intake.^(47)^ Third, food intake is affected by a complex network of factors, such as social, economic, and cultural aspects, potentially affecting the generalizability of our findings to all patients with INAD globally. Despite these limitations, this is, to our knowledge, the first study to assess the nutritional status, dietary habits, and feeding characteristics of patients with INAD, underscoring the importance of addressing these factors within a holistic and multidisciplinary care approach.

## CONCLUSION

Infantile neuroaxonal dystrophy presents significant nutritional and clinical challenges for patients and their families. Dysphagia was highly prevalent and contributed to inadequate nutrition and impaired weight gain. These findings underscore the importance of coordinated medical, nutritional, and therapeutic interventions to address the multifaceted symptoms of infantile neuroaxonal dystrophy. In resource-limited settings, public policies must improve access to multidisciplinary teams to address these unmet needs.

Maintaining adequate nutritional status and dietary balance is critical for the health and well-being of patients with infantile neuroaxonal dystrophy. Further research is required to better understand the relationship between infantile neuroaxonal dystrophy progression, feeding difficulties, and nutritional deterioration, as well as to identify effective strategies for managing feeding-related complications and optimizing nutritional care.

## Data Availability

The data cannot be made publicly available. Please justify below: Individual-level data will not be made publicly available due to ethical considerations regarding participants’ privacy and confidentiality. As this study involves a rare disease and a population of children and adolescents, the combination of demographic and clinical characteristics may allow the direct or indirect identification of participants, even after the removal of personal identifiers.
